# Supplementary material for: Birds repurpose the role of drag and lift to take off and land
Source: Nat Commun. 2019 Nov 25;10:5354. doi: 10.1038/s41467-019-13347-3 (PMC6877630; doi:10.1038/s41467-019-13347-3)
Supplement: Supplementary file 1 — Supplementary Information [file 41467_2019_13347_MOESM1_ESM.pdf]

Supplementary Information

Birds repurpose the role of drag and lift to take off and land

Diana D. Chin and David Lentink

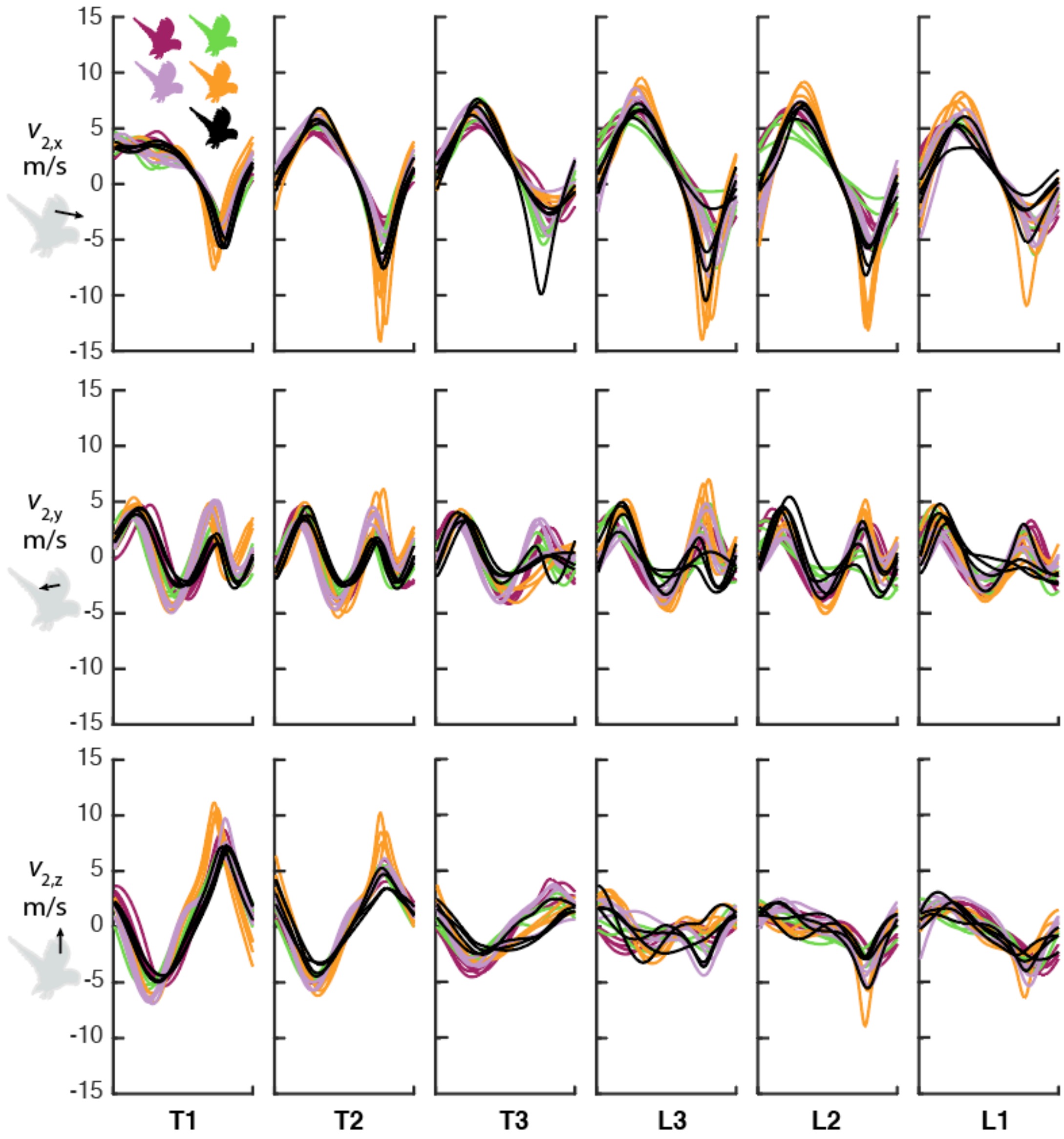

Supplementary Figure 1. Wing velocity components at the second moment of area *r_2*. Time traces of the *x*, *y*, and *z* wing velocity components at r_2 during the first three takeoff wingbeats (T1, T2, T3) and the final three full landing wingbeats (L3, L2, L1) are shown for all recorded flights. Colors represent the 5 individual parrotlets (colored traces correspond to the 4 pale blue parrotlets, and the black trace corresponds to the darker blue parrotlet).

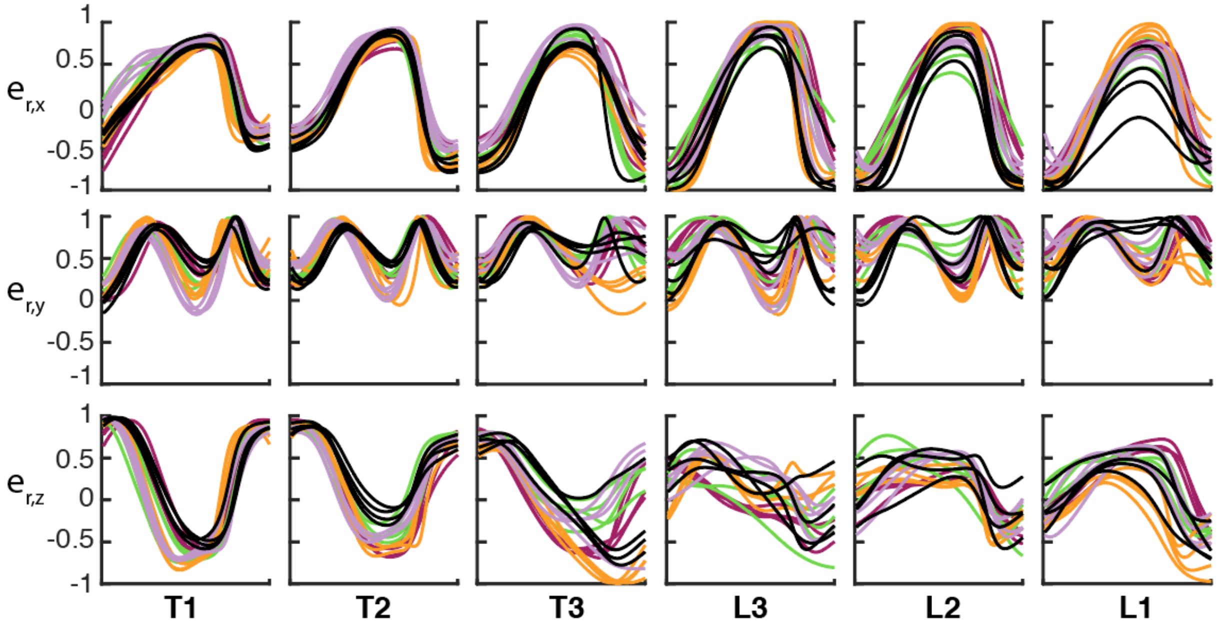

Supplementary Figure 2. Wing radius direction components during takeoff and landing wingbeats. Time traces of the parrotlets' wing radius direction ($\hat{\mathbf{r}} =< e_{r,x}, e_{r,y}, e_{r,z} >$) during takeoff (T1, T2, T3) and landing (L3, L2, L1) are shown for all recorded flights. Colors represent the 5 individual parrotlets (colored traces correspond to the 4 pale blue parrotlets, and the black trace corresponds to the darker blue parrotlet).
