# Supplementary material for: Birds repurpose the role of drag and lift to take off and land
Source: Nat Commun. 2019 Nov 25;10:5354. doi: 10.1038/s41467-019-13347-3 (PMC6877630; doi:10.1038/s41467-019-13347-3)
Supplement: Supplementary file 3 — Reporting Summary [file 41467_2019_13347_MOESM3_ESM.pdf]

nature research

Corresponding author(s): David Lentink, Diana D. Chin

Last updated by author(s): 2019/10/27

Reporting Summary

Nature Research wishes to improve the reproducibility of the work that we publish. This form provides structure for consistency and transparency in reporting. For further information on Nature Research policies, see Authors & Referees and the Editorial Policy Checklist .

Statistics

For all statistical analyses, confirm that the following items are present in the figure legend, table legend, main text, or Methods section.

n/a	Confirmed	
☐	☒	The exact sample size (*n*) for each experimental group/condition, given as a discrete number and unit of measurement
☐	☒	A statement on whether measurements were taken from distinct samples or whether the same sample was measured repeatedly
☒	☐	The statistical test(s) used AND whether they are one- or two-sided<br>*Only common tests should be described solely by name; describe more complex techniques in the Methods section.*
☒	☐	A description of all covariates tested
☒	☐	A description of any assumptions or corrections, such as tests of normality and adjustment for multiple comparisons
☐	☒	A full description of the statistical parameters including central tendency (e.g. means) or other basic estimates (e.g. regression coefficient) AND variation (e.g. standard deviation) or associated estimates of uncertainty (e.g. confidence intervals)
☒	☐	For null hypothesis testing, the test statistic (e.g. *F*, *t*, *r*) with confidence intervals, effect sizes, degrees of freedom and *P* value noted<br>*Give P values as exact values whenever suitable.*
☒	☐	For Bayesian analysis, information on the choice of priors and Markov chain Monte Carlo settings
☒	☐	For hierarchical and complex designs, identification of the appropriate level for tests and full reporting of outcomes
☒	☐	Estimates of effect sizes (e.g. Cohen's *d*, Pearson's *r*), indicating how they were calculated

Our web collection on statistics for biologists contains articles on many of the points above.

Software and code

Policy information about availability of computer code

Data collection	Data collection was done using LabVIEW 2016.
Data analysis	Data analysis was performed using custom code in Mathworks Matlab 2018b.

For manuscripts utilizing custom algorithms or software that are central to the research but not yet described in published literature, software must be made available to editors/reviewers. We strongly encourage code deposition in a community repository (e.g. GitHub). See the Nature Research guidelines for submitting code & software for further information.

Data

Policy information about availability of data

All manuscripts must include a data availability statement. This statement should provide the following information, where applicable:

- Accession codes, unique identifiers, or web links for publicly available datasets
- A list of figures that have associated raw data
- A description of any restrictions on data availability

The datasets generated and analyzed during the current study are available from the authors on reasonable request.

Field-specific reporting

Please select the one below that is the best fit for your research. If you are not sure, read the appropriate sections before making your selection.

☐ Life sciences ☐ Behavioural & social sciences ☒ Ecological, evolutionary & environmental sciences

For a reference copy of the document with all sections, see nature.com/documents/nr-reporting-summary-flat.pdf

Ecological, evolutionary & environmental sciences study design

All studies must disclose on these points even when the disclosure is negative.

Study description	We recorded voluntary, perch-to-perch flights made by 5 Pacific parrotlets (Forpus coelestis) inside of an aerodynamic force platform. Each parrotlet flew from the takeoff perch to the landing perch when cued by the trainer's finger. We recorded 4 flights per bird for a total of 20 birds.
Research sample	We tested 5 Pacific parrotlets (Forpus coelestis), 3 male and 2 female. We selected these birds because: (1) they are generalist birds, and thus should fly similarly to most small, arboreal birds; (2) they are relatively small, so we can record more wingbeats inside of the aerodynamic force platform, which could not be made larger without loss of temporal resolution; (3) they are more trainable, which we needed so that we could train and record voluntary perch-to-perch flights to best simulate foraging flight conditions.
Sampling strategy	We selected five birds, because this was the maximum number of birds that we were able to train to fly perch-to-perch inside of our experimental setup. Four flights were recorded for each bird to ensure that we would have sufficient data to find a mean and standard deviation across all birds within the scope of the study.
Data collection	The first author recorded the data from the aerodynamic force platform and high speed cameras, which were all connected to a computer for data collection.
Timing and spatial scale	All data were taken from the aerodynamic force platform, which has a flight volume of 100 x 100 x 60 cm (length x height x width). Most of the flights (18 out of 20) were recorded over the course of a few hours on September 19, 2018. One bird became reluctant to fly after two flights, so the final two flights were recorded the following morning (on September 20, 2018).
Data exclusions	No data were excluded from the analysis.
Reproducibility	We expect that this study should be reproducible to the extent that the same setup can still be used to get similar force recordings from these birds. We thoroughly tested and validated the setup before this study and also made similar recordings during a pilot study in which the recorded forces and kinematics were nearly identical to those presented in this study.
Randomization	We did not allocate the individuals into groups in our study, so no randomization was needed.
Blinding	Blinding was not relevant to our study, because we did not test different conditions between/among different groups.

Did the study involve field work? ☐ Yes ☒ No

Reporting for specific materials, systems and methods

We require information from authors about some types of materials, experimental systems and methods used in many studies. Here, indicate whether each material, system or method listed is relevant to your study. If you are not sure if a list item applies to your research, read the appropriate section before selecting a response.

Materials & experimental systems

n/a	Involved in the study	
☒	☐	Antibodies
☒	☐	Eukaryotic cell lines
☒	☐	Palaeontology
☐	☒	Animals and other organisms
☒	☐	Human research participants
☒	☐	Clinical data

Methods

n/a	Involved in the study	
☒	☐	ChIP-seq
☒	☐	Flow cytometry
☒	☐	MRI-based neuroimaging

Animals and other organisms

Policy information about studies involving animals; ARRIVE guidelines recommended for reporting animal research

Laboratory animals	Forpus coelestis, 3 male and 2 female, all approximately 6 years old.
Wild animals	The study did not involve wild animals.
Field-collected samples	The study did not involve samples collected from the field.
Ethics oversight	All training and experimental procedures were approved by Stanford's Administrative Panel on Laboratory Animal Care.

Note that full information on the approval of the study protocol must also be provided in the manuscript.
