# Supplementary material for: Birds repurpose the role of drag and lift to take off and land
Source: Nat Commun. 2019 Nov 25;10:5354. doi: 10.1038/s41467-019-13347-3 (PMC6877630; doi:10.1038/s41467-019-13347-3)
Supplement: Supplementary file 4 — Description of Additional Supplementary Files [file 41467_2019_13347_MOESM4_ESM.pdf]

Description of Additional Supplementary Files

File Name: Supplementary Movie 1
Description: Net forces during a foraging flight inside of the aerodynamic force platform. The total net force is shown in black, the net lift force is shown in blue, and the net drag force is shown in red. Forces scale with arrow length, with 1 bodyweight shown in green for reference.
